# Identifying Potential Drug-Related Problems Among Geriatric Patients With Use of an Integrated Clinical Decision Support Tool

**DOI:** 10.3389/fphar.2022.761787

**Published:** 2022-03-28

**Authors:** Veera Bobrova, Daniela Fialová, Shane Desselle, Jyrki Heinämäki, Daisy Volmer

**Affiliations:** ^1^ Faculty of Medicine, Institute of Pharmacy, University of Tartu, Tartu, Estonia; ^2^ Department of Social and Clinical Pharmacy, Faculty of Pharmacy in Hradec Králové, Charles University, Prague, Czechia; ^3^ Department of Geriatrics and Gerontology, First Faculty of Medicine, Charles University, Prague, Czechia; ^4^ Touro University California College of Pharmacy, Vallejo, CA, United States

**Keywords:** drug related problems, Estonia, multi-morbidity, older adults, polypharmacy, potentially inappropriate medications, clinical decision support tool

## Abstract

**Background:** Drug-related problems (DRPs) which arise from potentially inappropriate medications (PIMs) are a common problem in older people with multi-morbidity and polypharmacy.

**Aim:** To develop an integrated PIM clinical decision support tool for identification of DRPs in geriatric multi-morbid polypharmacy patients, using the EU(7)-PIM and EURO-FORTA lists, with a focus on high-risk medications.

**Methods:** The integrated PIM tool used the information on PIMs in both databases—the EU(7)-PIM and EURO-FORTA. PIMs were classified into four color groups based on risk profile: high-risk PIMs (should be avoided in older patients) as red, moderate-risk PIMs (require dose and/or treatment duration adjustment) as yellow, low-risk PIMs (low DRP risk) as green, and questionable PIMs (incomplete/missing information) as grey.

**Results:** The summarized list of the high-risk (red and some grey) PIMs contained 81 active substances and medication classes. According to the ATC classification, most of the high-risk PIMs (n = 60, 74.1%) belong to the A, C, and N medication groups and 50.6% (n = 41) of the high-risk PIMs have currently marketing authorization in Estonia. The preliminary list of the moderate- and low-risk (yellow, green, and other grey) PIMs contained 240 active substances and medication classes, but sub-classification of this category into one or another group depends mainly on an individual patient´s clinical characteristics in a concrete analyzed study sample and needs further research.

**Conclusion:** The integrated clinical decision support tool based on the EU(7)-PIM and EURO-FORTA criteria addresses the need for more efficient identification of DRPs. It can be applied to identify PIMs and geriatric prescribing problems in different health care settings, and also in a context of little clinical information available.

## Introduction

As the world’s population ages, the proportion of older patients in the population potentially vulnerable to multi-morbidity and polypharmacy increases. In addition, various psychosocial problems including lack of social support and economic problems may further exacerbate the risk of poorer health and worsened quality of life (Lau and Dolovich, 2005; Crealey et al., 2012; Alldred et al., 2016; Cheen et al., 2019; Crutzen et al., 2019; Gudi et al., 2019; Šola et al., 2020). Polypharmacy and inappropriate medication use among geriatric populations particularly in Central and Eastern Europe requires more attention in various health care settings and should be specifically recognized and managed at the governmental level (Botev, 2012).

In Estonia, the proportion of older adults (65 years old and older) gradually increases, being at the moment 19.4% (Tuula et al., 2021). By the year 2050, it is expected to increase up to 27.9% (Statistics Estonia: Stati, 2021). After 30 years there will be a relatively high proportion of older people with an expected higher degree of chronic morbidity and potential polypharmacy. The need for geriatric care will increase even more (Sepp et al., 2021).

The e-health system in Estonia is known to be one of the most ambitious and advanced digital solutions in Europe, where more than 95% of the data provided by health care institutions has been digitized (E-Estonia: healthcare, 2020). Yet, there are still many challenges ahead to find new possibilities of integrating innovative solutions focused on specific population groups into the national e-health care system. More attention should be paid to the geriatric population’s rational drug prescribing and use, as there is still no universally integrated age-oriented e-health system in Estonia for monitoring potential drug-related problems (DRPs) and for reporting older patients’ outcomes.

For the purpose of safe and effective medication prescribing in older adults, several explicit and implicit assessment tools have been created to identify potentially inappropriate medications (PIMs) and drug-drug interactions (DDIs), along with other types of prescribing problems (Bala et al., 2019; Pazan et al., 2019; Reeve, 2020; Rantsi et al., 2021). Over the last few years, the European Union EU(7)-PIM list (Renom-Guiteras et al., 2015) and European “Fit fOR The Aged” (EURO-FORTA) list (Pazan et al., 2018) for older patients, as well as the STOPP/START criteria version 2 (O'Mahony et al., 2015), have been published as the latest (2015, 2018, and 2015, respectively) inappropriate geriatric prescribing evaluation tools more specific for Europe. Recent studies demonstrated that some PIM criteria are less sensitive when used separately (Fialova et al., 2005; Siebert et al., 2013; Elseviers et al., 2014; Reich et al., 2014), and thus the authors suggest integrating at least two PIM screening tools to increase the sensitivity for the identification of DRPs in geriatric patients. The EU(7)-PIM list was created as a screening tool for pharmacoepidemiological applications with minimal clinical information about the individuals concerned. It is also one of the very few PIM checklists that include suggestions for dose adjustments and therapeutic alternatives (Renom-Guiteras et al., 2015; Thummar et al., 2019). However, it does not take into account an important aspect such as the aims of the treatment, and it is mainly focused on the overtreatment of the geriatric patient (Renom-Guiteras et al., 2015). Thus, prescribing appropriateness for PIMs could be additionally addressed by using other criteria, e.g., the EURO-FORTA criteria that strongly relies on 26 main treatment indication groups (Pazan et al., 2018). The EURO-FORTA list addresses aspects of drug selection for diagnoses and both aspects of inappropriate drug treatment in older adults: overtreatment and undertreatment. In addition, the EURO-FORTA criteria contain beneficial medications for certain indications (Pazan et al., 2018; Curtin et al., 2019).

The aim of this study was to develop an integrated PIM clinical decision support tool for identification of DRPs in geriatric multi-morbid patients in Estonia, using the combination of existing European PIM tools: the EU(7)-PIM and EURO-FORTA lists with a special focus on high-risk medications in older patients.

## Materials and Methods

### Tool Selection

In this method study, the EU(7)-PIM (Renom-Guiteras et al., 2015) and EURO-FORTA lists (Pazan et al., 2018) were selected as the basis for preparing an integrated e-health clinical decision support tool for screening adverse drug reactions (ADRs), DDIs, and PIMs in Estonia. The EU(7)-PIM and the EURO-FORTA tools have different approaches to the PIM identification (Renom-Guiteras et al., 2015; Wehling, 2016; Pazan et al., 2018; Pazan et al., 2019), which thus lend well to their being integrated for even greater efficiency and effectiveness (Table 1). At the moment, there is no universal concept used in Estonia to evaluate the rationality and safety of drug use targeted specifically to the geriatric population. The EU(7)-PIM and the EURO-FORTA tools were selected, as these are designed to evaluate medicines regularly used in European countries. In the future, this concept may become a substantial part of the primary health care settings in Estonia.

**TABLE 1:** Short comparison of the explicit criteria-based EU(7)-PIM^1^ and EURO-FORTA^2^ tools.

	The EU(7)-PIM tool	The EURO-FORTA tool
Year	2015	2018
Number of experts; number of countries/regions involved	30; 7	64; 7
Mean Delphi consensus coefficient	0.9	0.9
Target population	older people ≥65 years	older people ≥65 years; or ≥60 years with ≥6 medications, ≥3 diagnoses
Number of active substances or drug classes	282 chemical substances or medication classes from 34 therapeutic groups	264 chemical substances or medication classes organized into 26 categories according to diagnosis or clinical syndrome
PIM identification	Class A: active substance (PIM) should be avoided in older adults	Class A: indispensable medication, clear-cut benefit
	Class B: active substance is PIM in case of certain clinical conditions/co-morbidities or active substance is only considered as PIM	Class B: medication with proven or obvious efficacy in older adults, but limited extent of effect and/or safety concerns
	Combination of class A and B	Class C: medication with questionable efficacy/safety profiles in the older adults which should be avoided or omitted; explore alternatives
		Class D: avoid if at all possible in older adults, omit first and use alternative substance
Specifications	Explicit	Has both implicit and explicit measures^3^ ^,^ ^4^
	Drug oriented listing approach	Patient-in-focus listing approach
	Often restricted to doses or treatment duration	Not restricted to doses or treatment duration
	Not related to specific illnesses or conditions (no drug–disease aspect)	Related to specific illnesses or conditions (drug–disease aspect)
	Has suggestions for dose adjustments and therapeutic alternatives	Does not suggest dose adjustments and therapeutic alternatives
	Suitable for pharmacoepidemiological applications	Suitable for pharmacoepidemiological applications

PIM—potentially inappropriate medication.

1 Renom-Guiteras A, Meyer G, Thürmann, PA. The EU(7)-PIM list: a list of potentially inappropriate medications for older people consented by experts from seven European countries. Eur J Clin Pharmacol. 2015; https://doi.org/10.1007/s00228-015-1860-9.

2 Pazan F, Weiss C, Wehling M. The EURO-FORTA (Fit fOR The Aged) list: International consensus validation of a clinical tool for improved drug treatment in older people. Drugs Aging. 2018; https://doi.org/10.1007/s40266-017-0514-2.

3 Pazan F, Kather J, Wegling M. A systematic review and novel classification of listing tools to improve medication in older people. Eur J Clin Pharmacol. 2019; https://doi.org/10.1007/s00228-019-02634-z.

4 Wehling, M. How to use the FORTA (“Fit fOR The Aged”) list to improve pharmacotherapy in the elderly. Drug Res. 2016; https://doi.org/10.1055/s-0035-1549935

As no clinical trials and animal tests were performed, nor sensitive personal data were used in the present study, it was not necessary to seek the approval of the Ethics Committee.

### Definition of the Color Indicators

**FIGURE 1:**
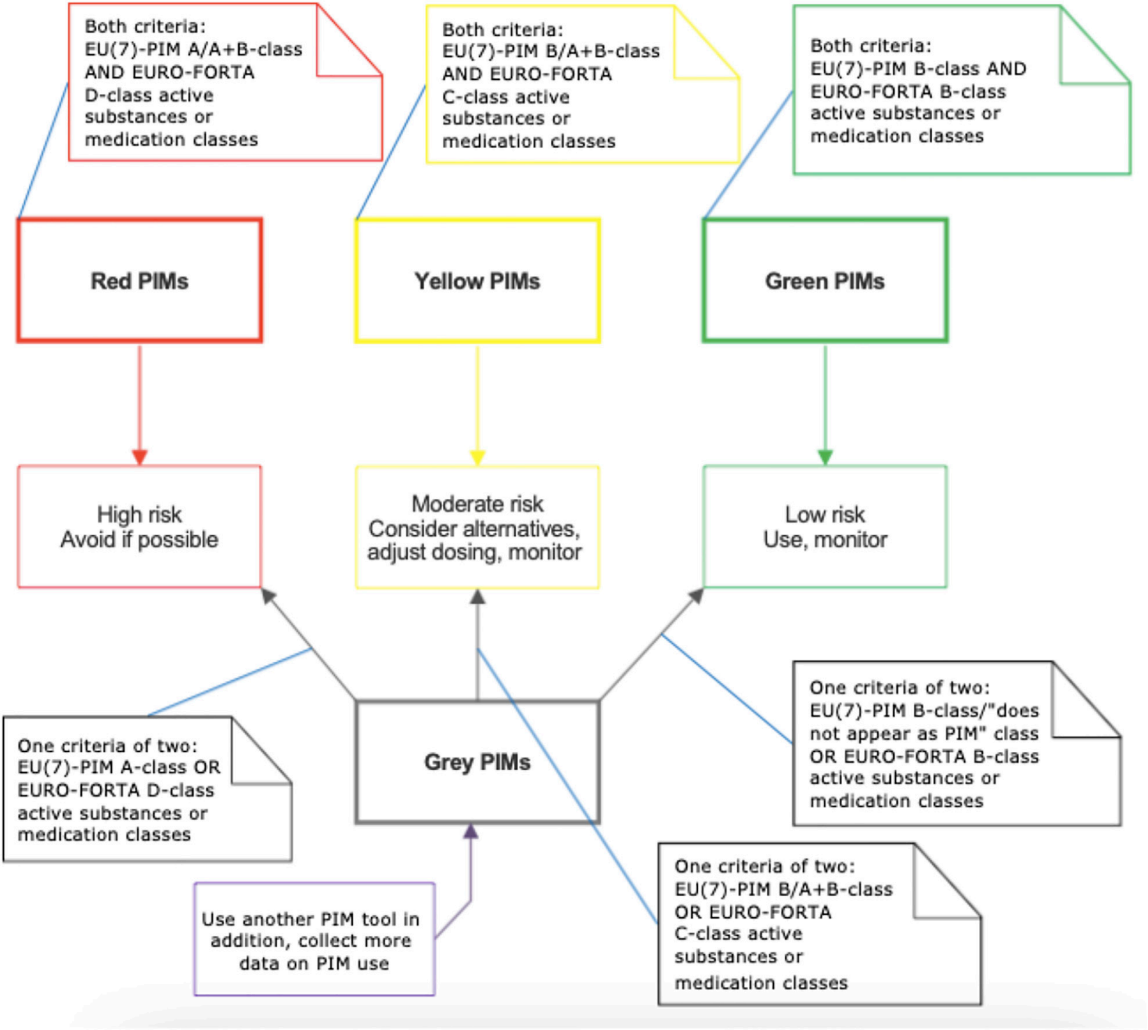
The classification of potential inappropriate medications (PIMs) according to the integrated screening PIM tool based on the EU(7)-PIM^1^ and EURO-FORTA^2^ lists.

Based on the risk and severity of potential adverse events described in the EU(7)-PIM and EURO-FORTA lists, the PIMs were suggested to be classified into four general color coding groups:

1) very significant PIMs as red color PIMs: active substances or medication classes that should be avoided in geriatric patients, when possible, and alternative treatment must be strongly considered (*high risk*);
2) significant PIMs as yellow color PIMs: active substances or medication classes that require mostly dose and/or treatment duration adjustment according to the patient health status and other medical details (*moderate risk*);
3) non-significant PIMs or non-PIMs as green color PIMs: active substances or medication classes that could be used in case of adequate therapy monitoring, older patients are not at potential high risk of DRPs (*low risk*);
4) questionable PIMs as grey color PIMs: any of the EU(7)-PIM or EURO-FORTA active substances or medication classes are presented whether in the EU(7)-PIM or in EURO-FORTA list only, and more data about the use of the particular PIM must be collected, or other PIM tool should be considered. The grey color PIMs can refer to all three risk profiles (high, moderate, and low). See Figure 1; Table 2 for more details.

**TABLE 2:** Detailed description of the integrated screening tool based on the EU(7)-PIM^1^ and EURO-FORTA^2^ lists.

	Type of PIMs	Description	Actions to be undertaken
High risk	Red color	Active substances or medication classes that refer to both the EU(7)-PIM *A-* or *A + B-class* and EURO-FORTA *D-class*	Avoid in older individuals, if possible, monitor patient safety, strongly consider alternative treatment. Only for grey color: collect more data on the PIM use, consider another PIM tool
	Grey color	Active substances or medication classes that refer to only the EU(7)-PIM *A-class* or to the EURO-FORTA *D-class*	
Moderate risk	Yellow color	Active substances or medication classes that refer to both the EU(7)-PIM *B- or A + B-class* and EURO-FORTA *C-class*, for the majority when used in higher doses and/or for longer treatment course than recommended in geriatric patients	Monitor patient safety, collect additional patient health data, consider dose adjustment, consider alternative treatment. Only for grey color: collect more data on the PIM use, consider another PIM tool
	Grey color	Active substances or medication classes that refer to only the EU(7)-PIM *B- or A + B-class* or to the EURO-FORTA *C-class*, for the majority when used in higher doses and/or for longer treatment course than recommended in geriatric patients	
Low risk	Green color	Active substances or medication classes that refer to both the EU(7)-PIM *B-class and EURO-*FORTA *B-class*, those with limited concerns on the effect or safety in geriatric patients or not considered as inappropriate when used in lower doses and/or for short treatment course in geriatric patients	Monitor treatment safety, repeat medication review on regular basis, patient is more likely not at the high risk of DRPs. Only for grey color: collect more data on the PIM use, consider another PIM tool
	Grey color	Active substances or medication classes that refer to only the EU(7)-PIM *B-class or marked as “does not appear as PIM” or to the EURO-*FORTA *B-class*, those with limited concerns on the effect or safety in geriatric patients or not considered as inappropriate when used in lower doses and/or for short treatment course in geriatric patients	

PIM—potentially inappropriate medication; DRP—drug related problem.

1 Renom-Guiteras A, Meyer G, Thürmann, PA. The EU(7)-PIM list: a list of potentially inappropriate medications for older people consented by experts from seven European countries. Eur J Clin Pharmacol. 2015; https://doi.org/10.1007/s00228-015-1860-9.

2 Pazan F, Weiss C, Wehling M. The EURO-FORTA (Fit fOR The Aged) list: international consensus validation of a clinical tool for improved drug treatment in older people. Drugs Aging. 2018; https://doi.org/10.1007/s40266-017-0514-2.

The content and structure of the present integrated PIM identification dataset based on the EU(7)-PIM and EURO-FORTA lists were defined in repeated sessions, including evaluation by experts from Estonia and the Czech Republic in the period April 2020 to May 2021. These experts are also contributing to the scientific works on the EUROAGEISM H2020 ESR7 project (2017-2021) entitled “Inappropriate prescribing and availability of medication safety and medication management services in older patients in Europe and other countries”.

### High-Risk (Red and Some Grey) Potentially Inappropriate Medications

In the present study, the authors focused on the high-risk PIMs: active substances or medication classes that are always (independently on individual clinical conditions) clinically very significant PIMs in older patients. According to the original PIM criteria used, both A-class EU(7)-PIM and D-class EURO-FORTA active substances or medication classes are those that should not be used in geriatric patients in general, as these can often bring potential medication-related harm.

The high-risk PIMs were developed as follows:

- all A-class active substances or medication groups were extracted from the EU(7)-PIM list with additional information about the reasoning of PIM classification and special considerations of use;
- the EURO-FORTA tool was screened for the same active substances or medication groups, and the respective class (A-, B-, C- or D-class) was specified for each PIM; in case of missing data, the EURO-FORTA class of the active substance or medication group was marked as “no information”;
- the rest of the EURO-FORTA D-class active substances or medication groups were extracted from the list and compared to the EU(7)-PIM criteria, and the respective class (A-, A + B-, B-class or “does not appear as PIM” class) was specified for each PIM; in case of missing data, the EU(7)-PIM class of the active substance or medication group was marked as “no information”;
- if the abovementioned active substances or medication classes are presented in both the EU(7)-PIM and EURO-FORTA criteria, the red color was used to indicate the high risk for the geriatric population. If the active substances or medication classes presented whether in the EU(7)-PIM or in EURO-FORTA list only, the grey color was used to highlight the need to collect more data about the use of these high-risk PIMs in older adults;
- the local (Estonia) and international (European Medicines Agency) Summaries of Product Characteristics (SmPCs) were used to collect any additional information on the use of PIMs in geriatric patients (e.g., dosage and treatment duration adjustment in older adults);
- the PIMs were checked for availability and actual use in Estonia by addressing the official register of medications (2021) (State Agency of Medicines, 2019; State Agency of Medicines, 2020).

### Moderate- and Low-Risk (Yellow, Green, and Other Grey) Potentially Inappropriate Medications

The process of identifying the moderate- and low-risk PIMs according to the integrated PIM tool depends directly on individual patient characteristics and many factors concerning the patient´s health status and other clinical issues. For most of the yellow and green PIMs, the clinical relevance of a particular PIM may change depending on the duration of treatment and dosing, treatment indication, and possible DDIs and therapeutic duplications. These PIMs should be considered when the treatment rationality at an individual patient level is assessed in geriatric patients. For this reason, for the moderate- and low-risk PIMs a preliminary list was prepared. The creation of the preliminary list leaves the matter of the moderate- and low-risk PIMs partly open and allows subsequent modifications of the list in the future, if needed. The list of the moderate- and low-risk PIMs was developed as follows:

- all B- and C-class active substances or medication groups (excluding those with the high risk) were extracted from the EURO-FORTA list;
- the EU(7)-PIM tool was screened for the same active substances or medication groups with additional information about reasoning of PIM classification and special considerations of use, and the respective class (A + B- and B-class or “does not appear as PIM” class, excluding A-class referred to the high risk) was specified for each PIM;
- where possible, the risk (moderate or low) and the color (yellow, green, or grey) were established for each individual active substance or medication group according to the integrated method. In addition, the factors that could potentially affect the actual risk for older patient (e.g., adverse events, dosing, duration of treatment, indication, renal functions) were specified based on the EU(7)-PIM and EURO-FORTA criteria;
- the rest of the active substances or medication groups, for which it was difficult to predict the risk due to the missing information in one of the PIM criteria, formed the “Other potential moderate- or low-risk PIMs” group with the need for future research.

By this, the moderate- and low-risk PIMs are all those PIMs mentioned in the EU(7)-PIM and EURO-FORTA lists, that are not treated as high-risk PIMs according to the present study methodology. The active ingredients or medication classes are categorized as yellow not only because of the active substance itself (like most of the red color PIMs) but in many cases due to the long-term treatment course and high doses being inappropriate for geriatric patients. In contrast, green color PIMs could be mostly appropriate for geriatric patients when used in lower doses and/or for a shorter period of time, as stated in the EU(7)-PIM and EURO-FORTA criteria. Still, there could be always some exceptional cases in clinical practice, e.g., when the green color PIM can become inappropriate or classified as yellow color PIM. Other moderate- or low-risk **(**grey color) PIMs are those with missing data in the integrated PIM tool that are not possible to classify as yellow or green color PIMs, and the risk (moderate or low) cannot be specified in general. For these PIMs, there is a need to use additional data sources (e.g., any other PIM list).

## Results

### High-Risk (Red and Some Grey) Potentially Inappropriate Medications

**FIGURE 2:**
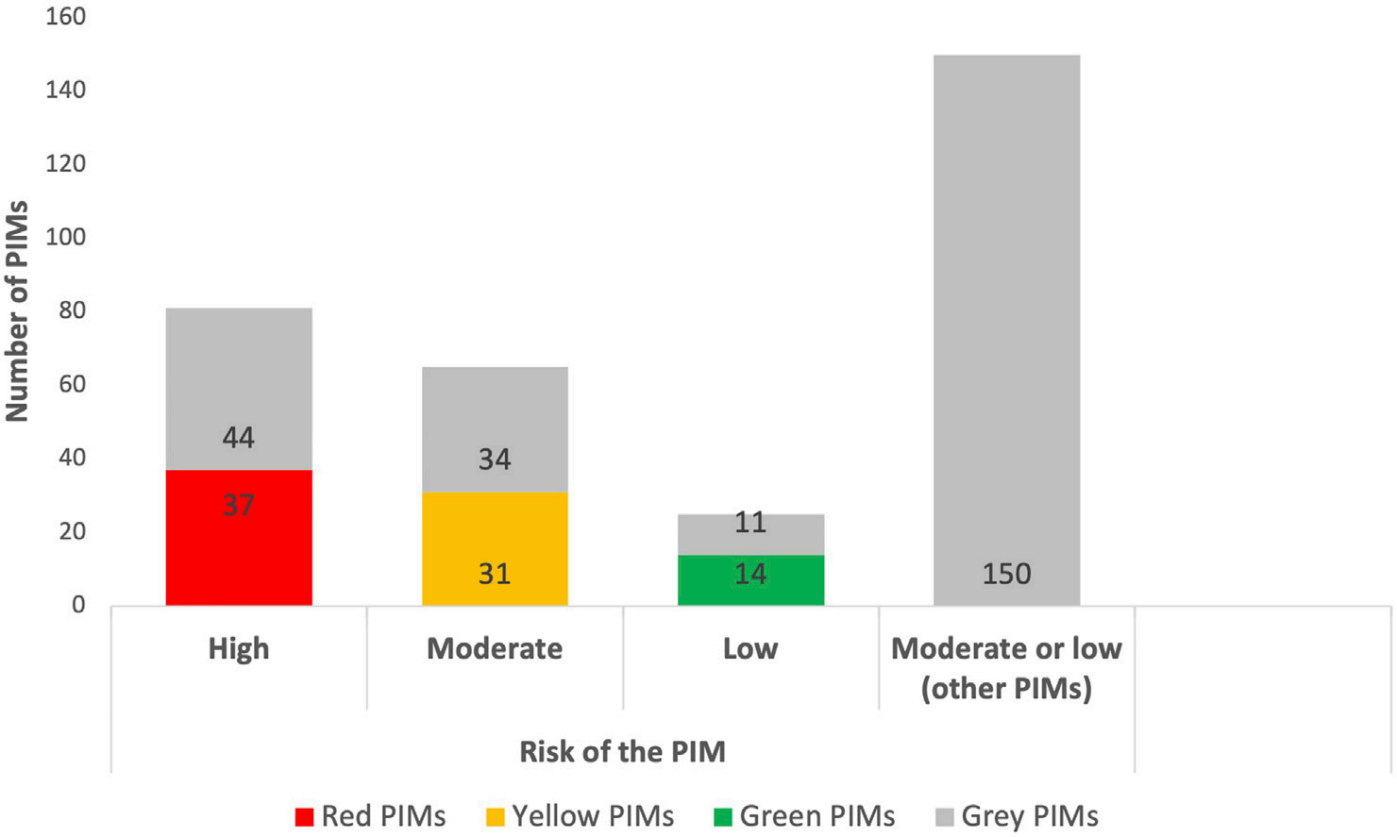
Proportion of the high-, moderate- and low-risk PIMs in the integrated screening tool based on the EU(7)-PIM^1^ and EURO-FORTA^2^ criteria.

According to the integrated PIM clinical decision support tool, the total list of the high-risk PIMs contained 81 active substances, including one combination of two medications, eight medication classes and two classes of dietary and other oral Supplementary Appendix S1. Of the PIMs identified, there were 37 (45.7%) red and 44 (54.3%) grey color high-risk PIMs in the tool (Figure 2).

The study methodology suggests that 61 (75.3%) of the high-risk PIMs originally belonged to the EU(7)-PIM criteria, and the same number of the high-risk PIMs originally belonged to the EURO-FORTA criteria. Half of the high-risk PIMs (n = 41, 50.6%) belonged to both criteria at the same time. Thus, the present integrated PIM tool consists of 41 high-risk PIMs that present in the EU(7)-PIM and EURO-FORTA criteria (coinciding PIMs), 20 high-risk PIMs that present only in the EU(7)-PIM, and 20 high-risk PIMs that present only in the EURO-FORTA. Therefore, the tool enables to identify 20 high-risk PIMs more (81 PIMs) than the EU(7)-PIM (61 PIMs) and EURO-FORTA (61 PIMs) can determine if used separately (Table 3).

**TABLE 3:** High-risk PIMs with and without marketing authorization in Estonia (n = 81, 100%).

	All high-risk PIMs% (n)	High-risk PIMs with marketing authorization in Estonia% (n)	High-risk PIMs not authorized but still marketed in Estonia (ET, RT)% (n)	High-risk PIMs not authorized and not marketed in Estonia% (n)
PIMs listed in the integrated tool	100 (81)	50.6 (41)	16.1 (13)	33.3 (27)
Originally belong to the EU(7)-PIM criteria^1^	75.3 (61)	38.3 (31)	11.1 (9)	25.9 (21)
Originally belong to the EURO-FORTA criteria^2^	75.3 (61)	40.7 (33)	14.8 (12)	19.8 (16)
Originally belong to both criteria at the same time^1^ ^,^ ^2^	50.6 (41)	28.4 (23)	9.9 (8)	12.3 (10)
Red PIMs	45.7 (37)	26.0 (21)	8.6 (7)	11.1 (9)
Grey PIMs	54.3 (44)	24.7 (20)	7.4 (6)	22.2 (18)
A (alimentary tract and metabolism) ATC group	18.5 (15)	8.65 (7)	1.2 (1)	8.65 (7)
C (cardiovascular system) ATC group	22.2 (18)	8.65 (7)	4.9 (4)	8.65 (7)
G (genito urinary system and sex hormones)	3.7 (3)	3.7 (3)	0	0
J (antiinfectives for systemic use) ATC group	1.2 (1)	1.2 (1)	0	0
L (antineoplastic and immunomodulating agent)	1.2 (1)	1.2 (1)	0	0
M (musculo-skeletal system) ATC group	7.4 (6)	2.5 (2)	1.2 (1)	3.7 (3)
N (nervous system) ATC group	33.3 (27)	18.5 (15)	6.2 (5)	8.6 (7)
R (respiratory system) ATC group	8.8 (7)	3.7 (3)	1.2 (1)	3.7 (3)
V (various)	1.2 (1)	0	1.2 (1)	0
Geriatric information in SmPC was found	49.4 (40)	34.6 (28)	6.2 (5)	8.6 (7)

ATC, Anatomical Therapeutic Chemical (classification); ET (Erialaorganisatsiooni Taotlusega ravimid, est) and RT (Ravimiameti Taotlusega ravimid, est): used by application of specialized physician, hospitals or research institutions; PIM, potentially inappropriate medication; SmPC, Summaries of Product Characteristics.

1 Renom-Guiteras A, Meyer G, Thürmann, PA. The EU(7)-PIM list: a list of potentially inappropriate medications for older people consented by experts from seven European countries. Eur J Clin Pharmacol. 2015; https://doi.org/10.1007/s00228-015-1860-9.

2 Pazan F, Weiss C, Wehling M. The EURO-FORTA (Fit fOR The Aged) list: International consensus validation of a clinical tool for improved drug treatment in older people. Drugs Aging. 2018; https://doi.org/10.1007/s40266-017-0514-2.

Most of the high-risk PIMs belonged to the N (nervous system), C (cardiovascular system), and A (alimentary tract and metabolism), medication groups according to the ATC (Anatomical Therapeutic Chemical) classification (Table 3). For half (n = 40, 49.4%) of the high-risk PIMs, the authors found specific information in SmPC about use in geriatric patients (Table 3; Supplementary Appendix S1).

The number of the authorized and marketed high-risk PIMs in Estonia in 2021 was 50.6% (*n* = 41) from the total number of the corresponding PIMs in the developed integrated tool. This type of PIMs was mostly available as prescription (Rx, *n* = 30, 37.0%), but also as over-the-counter (OTC, n = 8, 9.9%) medications or dietary supplements (n = 3, 3.7%) (Table 3, Supplementary Appendix S1). The list of the high-risk PIMs that are not authorized in Estonia, but still used by the application of specialized physician, hospitals, or research institutions, consists of 13 active substances, which corresponds to 16.1% of the total number of the PIMs in the developed integrated tool. It was found that 27 (33.3%) of the high-risk PIMs were not authorized and not marketed in Estonia in 2021 (Table 3, Supplementary Appendix S1).

### Moderate- and Low-Risk (Yellow, Green, and Other Grey) Potentially Inappropriate Medications

As the identification of the yellow and green PIMs depends directly on an individual patient’s clinical characteristics, the authors of the present study prepared the preliminary list of the moderate- and low-risk PIMs, consisting of a total of 240 active substances or medication groups (Supplementary Appendix S2). The sub-classification and the proportion of the moderate- and low-risk PIMs in the integrated tool can be found from Supplementary Appendix S2; Figure 2.

The risk information presented in both EU(7)-PIM and EURO-FORTA tools enabled the authors to presume that 65 PIMs could be classified as moderate-risk PIMs: 31 (47.7%) being classified as yellow, and 34 (52.3%) as grey color PIMs. At the same time, 25 PIMs could be more likely associated with the low risk: 14 (56.0%) classified as green and 11 (44.0%) as grey color PIMs.

All 65 moderate-risk PIMs belong originally to the EURO-FORTA tool B- or C-class, and only 30 (46.2%) to the EU(7)-PIM A + B- and B-class, or to the “does not appear as PIM” class. Analogously, all 25 low-risk PIMs belong originally to the EURO-FORTA tool B-class and only 8 (32.0%) to the EU(7)-PIM B-class or “does not appear as PIM” class.

The rest 150 active substances or medication groups were defined as “other potential moderate- or low-risk PIMs”, where 126 (84.0%) PIMs originally belonged to the EU(7)-PIM list and 24 (16%) to the EURO-FORTA criteria (Supplementary Appendix S2).

Based on the EU(7)-PIM list, nine moderate-risk, and five low-risk active substances or medication groups were suggested as an alternative to some PIMs, and thus the authors of the present study marked them as “beneficial medications” (Supplementary Appendix S2). For this reason, the low-risk beneficial medications have the potential to be excluded from the list of the PIMs in the integrated tool as non-PIMs after validation of the tool. At the same time, the moderate-risk beneficial medications could be transferred to the low-risk PIM group after additional research.

## Discussion

This is the first study in Estonia focusing on the use of the EU(7)-PIM and EURO-FORTA criteria jointly to create an integrated PIM clinical decision support tool identifying potentially inappropriate prescribing for geriatric patients. Previous research has demonstrated that it is not sufficient to use only one PIM criteria in the study design, because it may give inconclusive results (Tommelein et al., 2016; Wamil et al., 2019; Johansen et al., 2020). The integrated PIM tool allows for a more specific assessment of the risks of PIMs and therefore enables it to become the convenient instrument to evaluate the complex medication use problems in primary health care settings. During the process of creation of the integrated PIM tool, the authors identified several important considerations that will be addressed and discussed below in the text along with some recommendations for future research.

The integrated PIM tool enabled to list of 25% more high-risk PIMs than the two separate tools (EU(7)-PIM and EURO-FORTA). In addition, a comparison of the two tools provided extended information on moderate- and low-risk PIMs, but also highlighted the lack of data on the use of PIMs in the elderly. In this study, a total of 194 PIMs (150 related to moderate- and low-risk PIMs) were identified with insufficient information for final classification. In order to identify the actual risk for these particular PIM, it is not sufficient to use the combination of the EU(7)-PIM and EURO-FORTA criteria, but also it is important to know some specific additional information on patients’ medication and clinical data, that may vary depending on the PIM being under examination. Future research is needed to identify described discrepancies that may differ depending on the specific country and local drug prescribing traditions and guidelines. At the same time, although the list of the high-risk PIMs and the preliminary list of the moderate- and low-risk PIMs were accepted unanimously by all authors of the present study, the PIMs with insufficient risk information always need additional inspection and consideration by experts in the field. It is conceivable that for these PIMs that could be classified as high-, moderate- or low-risk PIMs only by one of the lists [the EU(7)-PIM or EURO-FORTA list], there should be an even more detailed explanation on how or whether to use them in older adults.

The present study showed that the actual use of some high-risk PIMs in Estonia differs from the concept provided by the EU(7)-PIM and EURO-FORTA lists. For example, use of PIMs in combined medicinal products (dextromethorphan, diphenhydramine, estrogen, magnesium hydroxide); as OTCs and food supplements with different requirements to the patient information (Aloe, Ginkgo folium, magnesium hydroxide, *Senna* glucosides, and St. John’s Wort), and in a different pharmaceutical formulation (niacin - nicotinic acid - only as an injection, viscous paraffin, and minoxidil as external products). The study results suggest a need to further explore the problem of combined medicinal products and other discrepancies mentioned above before the integrated PIM tool becomes a widely available instrument for clinical use, and this suggestion is corroborated by the implications discussed in other studies (Sönnerstam et al., 2017; Curtin et al., 2019; Fialová et al., 2019). Another issue that should be addressed in the near future is the urgent need to update the PIM lists on a regular basis by inserting newly identified PIMs or changing the content of already existing PIMs (Wauters et al., 2016; European Monitoring Centr, 2019).

The study showed that the information concerning the rational and safe use of the medications in older adults was found from the SmPCs for only 49.4% (n = 40) of the high-risk PIMs. The additional information concerning safety aspects (e.g., dosage and treatment duration adjustment, or any other recommendations for the geriatric patients) must be collected, including appropriate medication safety studies. There are 16.1% (n = 13) of the high-risk PIMs with no marketing authorization in Estonia but still used in some Estonian patients/groups of patients by application of specialized physician, hospitals, or research institutions. The list of described high-risk PIMs can change many times a year depending on the necessity for the medications that are not presented in the country-specific drug market and thus closer attention should be paid to this group of PIMs. The authors see an urgent need to discover possible new PIMs that are relevant for Estonia and that could be added to the integrated PIM list in the future.

At the moment, the existing international PIM tools are available in Estonia only as original research papers (e.g., PDF documents). This makes their use in everyday clinical practice inconvenient and also reduces awareness and usability among healthcare professionals. The integration of the clinical decision support PIM tool to the e-health system in Estonia is the expected future step. It will help more efficiently identify patients at risk and improve the safety and efficacy of drug prescribing to older adults. Current software packages do not screen geriatric risks of medications. The information available on the implementation of both instruments [the EU(7)-PIM and EURO-FORTA] should be examined in advance and, if necessary, taken into account when applying the integrated PIM tool in real clinical practice. The important aspects that should be carefully considered before applying the integrated PIM list in practice are, for example, the information on how to use the EURO-FORTA list in daily clinical practice based on experiences from clinical trials and the personal experiences (“a use algorithm for FORTA”) of the authors of the list (Wehling, 2016), or any relevant information about the practical use of the EU(7)-PIM tool by different research groups (Sönnerstam et al., 2017; Thummar et al., 2019). In addition, it is crucial to investigate the ways to include additional information about the PIMs listed in both criteria in the design of the integrated PIM tool, e.g., “positive” list of active substances or medication classes (A- and B-class medications) for certain indications by EURO-FORTA, as well as suggestions for dose and/or treatment duration adjustments (also in relation to hepatic and renal function), and therapeutic alternatives for PIMs based on the EU(7)-PIM list. Lastly, the authors discuss the future option to include recommendations concerning the use of a similar alternative PIM checklist [e.g., the Ghent Older People’s Prescriptions Community Pharmacy Screening (GheOP3S)‐Tool Version 2] for identification of the grey PIMs or any other possible discrepancies (Foubert et al., 2021).

As the concept (color coding) of the present integrated tool is intentionally similar to the drug interaction and counter indication decision support software based on the inxbase/riskbase database used in Estonia (Inxbase and Riskbase data, 2020), the tool can become a part of this software. It will be focused more on the older populations’ safe and rational medication use, and can also be applied in a context of little clinical information available. In this scenario, access to the integrated PIM tool may be provided in the future to health care employees from different care settings, including doctors, nurses, pharmacists, and others. For similar purposes, it could also be used in other countries which may benefit from applied methodology into the combination of these two internationally, widely recognized tools.

## Strengths and Limitations

The integrated PIM clinical decision support tool could support the process of detection of high-risk medications for older adults. It could also help to state more specific risks for each PIM compared to the use of either one of the individual PIM lists [e.g., the EU(7)-PIM or EURO-FORTA]. From both criteria used [e.g., the EU(7)-PIM or EURO-FORTA] as a basis in the design of the present integrated tool, the appropriate information on PIMs was adopted so that it helps to reach a full-fledged examination of DRPs in geriatric patients and to apply this approach in case of patients with limited clinical data. The integrated PIM tool is based on the structured color categorization of the PIMs by risk and severity of using them in older people and has both, drug-oriented and patient-in focus approaches. In addition to high-risk medications, the tool was designed to determine moderate- and low-risk PIMs, but in this case, sufficient patient clinical information will be needed. On the basis of what has been stated above, this is the real patient data that indeed plays a critical role in the process of PIM categorization for moderate- and low-risk PIMs. This situation forced the authors of the present study to abandon the idea to put together the complete list of moderate- and low-risk PIMs. Thus, the preliminary list of this type if PIMs was prepared with a future perspective for additional research in this area. The authors of the present study deem it appropriate to conduct the tool validation with real patients first in order to understand the actual need for the combined tool within healthcare employees in Estonia and to see if the tool and its concept is understandable and practical. Thus, the validation of the tool is the future step that must be undertaken before the tool can be implemented in real clinical practice. Although the tool is not yet validated it indicates the preliminary evidence of it identifying PIMs more germane in this context and shows promise in being piloted as an effective PIM tool in future clinical studies. It must be also acknowledged that the quality of the integrated tool is directly linked to the updating of the original PIM criteria and that this type of tools should be updated on a regular basis. And last but not least, each country or region should adapt the assessment PIM tools according to the medications on the market (including combined drugs) available and the traditions of their clinical use.

## Conclusion

This study introduces a novel integrated PIM clinical decision support tool based on the two European most widely known and used the EU(7)-PIM and EURO-FORTA criteria to address the need for more efficient identification of DRPs in geriatric, multi-morbid patients. The present integrated tool consists of 321 active ingredients or medication classes. Based on the information in the source instruments, there was the most background information for the classification of high-risk PIMs, enabling to recognize 25% more respective PIMs than with the EU(7)-PIM or EURO-FORTA separately. On the other hand, for detailed classification, approximately half of the high-risk PIMs and the majority of the moderate or low-risk PIMs require further information on the use of medicines in older adults, or the clinical and other characteristics of a particular patient. This result points to a continuing lack of information on the geriatric use of medicines, as well as the need to integrate the use of theoretical tools into everyday medical practice, especially in the context of polypharmacy growth. The validation of the integrated tool is the next step in its development and implementation in clinical practice.

## Data Availability

The original contributions presented in the study are included in the article/Supplementary Material, further inquiries can be directed to the corresponding author.

## Footnotes

1 Renom-Guiteras A, Meyer G, Thürmann, PA. The EU(7)-PIM list: a list of potentially inappropriate medications for older people consented by experts from seven European countries. Eur J Clin Pharmacol. 2015; https://doi.org/10.1007/s00228-015-1860-9.

2 Pazan F, Weiss C, Wehling M. The EURO-FORTA (Fit fOR The Aged) list: International consensus validation of a clinical tool for improved drug treatment in older people. Drugs Aging. 2018; https://doi.org/10.1007/s40266-017-0514-2.
